# Effect of COVID-19 on Orthopaedic Trauma Admissions and Operating in a London District General Hospital

**DOI:** 10.1055/s-0042-1757883

**Published:** 2022-10-10

**Authors:** Ubaid Zahoor, Catherine Malik, Hassan Raja, Sruthi Ramaraju, Kesavan Sri-Ram

**Affiliations:** 1 Trauma and Orthopedics Department, Whipps Cross Hospital, London, United Kingdom; 2 Imperial College London, London, United Kingdom

**Keywords:** COVID-19, pandemic, orthopaedic and trauma admissions, DGH

## Abstract

**Background** The coronavirus disease 2019 (COVID-19) has presented orthopaedic departments around the world with unprecedented challenges across all aspects of health care service delivery. This study explores the effect of the COVID-19 lockdown on trauma admissions and trauma theater utilization at a London District General Hospital.

**Methods** Data was collected retrospectively from electronic patient records for 4 weeks from the initiation of two lockdown periods beginning March 16, 2020 and December 23, 2020. Results were compared with a comparable time period in 2019. Patient age, date of admission, time of admission, date of operation, length of stay, length of operation, type of operation, and length of anesthesia were analyzed.

**Results** Fewer patients were admitted during the COVID-19 period for trauma (108 in 2019 vs. 65 in March 2020 and 77 in December 2020). In addition, there was a significant shift in patient demographics, with the mean age of patients being 55.6 years in 2019 and 64.1 years in March 2020 and December 2020 ( *p* = 0.038). The most common mechanism of injury in both years was due to falls; however, the proportion of injuries due to falls fell from 75% in 2019 to 62% March 2020, but not significant change from pre-COVID baseline in December 2020 (77% falls). The duration of anesthesia was significantly longer in March 2020 (136 minutes) compared with in 2019 (83 minutes) ( *p* < 0.00001). There was no statistically significant difference in operation length for each operation type, but there was an overall increase in median operation length of 13.6% in March 2020 from the previous year. Finally, although overall length of stay was roughly constant, the time between admission and operation was significantly reduced in March 2020 (1.22 vs. 4.74 days, *p* < 0.0000001).

**Conclusion** Orthopaedic trauma remains an essential service which has always had to overcome the challenges of capacity and resources in busy cities like London. Despite the reduction in trauma volume during the COVID-19 lockdown there have still been significant pressures on the health care system due to new challenges in the face of this new disease. By understanding the effects of the lifestyle restrictions brought about by the lockdown on trauma services as well as the impact of COVID-19 on service delivery measures such as length of surgery and stay, health care managers can plan for service delivery in the future as we attempt to return to nonemergency orthopaedic services and move lockdown restrictions are eased.

## Background

The COVID-19 has presented orthopaedic services around the world with unprecedented challenges across all aspects of health care service delivery. The first cases of COVID-19 in the U.K. were reported in late January; however, it was not until March 13 that the U.K. Health Secretary advised against all unnecessary social contact. Clinical decision making now had a new lens through which it was viewed, balancing optimum treatment of a patient's injury against clinical safety and resources, balancing the risks of exposure both for patients and staff. The Federation of Surgical Specialty Associations released guidance on the management of patients with urgent orthopaedic conditions and trauma in April 2020, 1 after initiation of lockdown measures in the U.K., which guided our surgical decision making in conjunction with patient engagement. Patient pathologies were stratified according to urgency of treatment in to P1, P2, P3, P4, and P5. P1 was further divided into P1a and P1b, with recommended operation times within 24 and 72 hours, respectively. P2 patients were recommended to be operated on within 1 month. During the initial lockdown of March 2020, it was recommended that only P1 and P2 operations should occur. At our center all patients presenting with injuries fulfilling these criteria were counseled on the risks and benefits of the operation and the likely sequelae of delay.

Whipps Cross University Hospital is a large 586-bed 2 District General Hospital (DGH) within Epping Forest in the London Borough of Waltham Forest. It is part of Barts Health National Health Service (NHS) Trust and orthopeadic services within the Trust also take place at Newham University Hospital and the Royal London Hospital. Orthopaedic operating capacity at Whipps Cross Hospital prior to the 2020 COVID-19 pandemic included two dedicated orthopaedic theaters with laminar flow in the main theater suite, one for the daily trauma list and the other for elective lists which would also include trauma when needed, as well as one orthopaedic day case theaters that could accommodate elective and trauma cases. To minimize exposure to staff and patients, and to reduce logistical and staffing pressures due to the rising number of COVID-19 patients, all elective orthopaedics surgery was cancelled after the 20th of March. The day-case unit was converted to a COVID high dependency unit (HDU) and intensive care unit (ITU) and trauma operating capacity was reduced to the emergency theaters only. Theater availability during the initial March 2020 lockdown consisted of one emergency theater for utilization by all specialities, and when staffing levels allowed one dedicated trauma theater. A separate theater was set aside for COVID-positive patients only. However, this was staffed by the same theater team as the emergency theater, so both theaters could not be running simultaneously. There were a handful of days where due to staff redeployment and staff illness/isolation there was only one emergency theater running to accommodate all surgical specialities.

NHS guidance for the management of orthopaedic patients during the COVID pandemic also recognized trauma services as a key service 3 that would continue to have large demand despite lockdown measures. Strategies to meet this demand while minimizing patient exposure in-hospital were also described. These guidelines included treating as many admissions as day-cases as possible, 3 for example, through reducing preoperative delays, and by choosing alternative surgical procedures that require less postoperative input where safe and possible to do so. In addition, emphasis was placed on nonoperative management of patients where possible. This left us with only emergency procedures which were absolutely necessary and those which would have significant morbidity if delayed. Patients were involved in the clinical decision making and some (not recorded in the scope of this study) declined surgical management.

In this analysis we aim to compare our trauma workload during two lockdown periods of the COVID-19 pandemic (March and December 2020), whereby social restrictions were similar, with a comparable pre-COVID time period from March 2019.

## Methods

### Selection of Time Periods

The two time periods during the COVID-19 pandemic were chosen based on similar social restrictions being in place, and similar staffing pressures within the hospital with high numbers of COVID-19-infected inpatients.

The first social lockdown in the U.K. was brought into effect on March 23, 2020. 4 This could be legally enforced from March 26, 2020. The social restrictions at this time were to stay at home under all circumstances except: essential work (key workers), essential travel, and 1 hour daily exercise avoiding contact with those outside your household. No mixing of households either inside or outside was permitted. These restrictions were then slowly eased over the coming months, beginning May 10.

A second 4-week national lockdown ensued from November 5. However, this was widely viewed as a pre-Christmas firebreak. Inpatient numbers of COVID-19 patients were not comparable to the initial lockdown period in March, and the majority of staff were able to remain within their usual roles, rather than be redeployed to COVID care.

On December 23, 2020 London was subjected to social restrictions similar to the initial March lockdown and the full impact of the second wave was becoming apparent. Staff again were redeployed and inpatient COVID-19-infected patients exceeded the March numbers in many places. 5

### Data Collection

Data was collected from electronic patient records retrospectively for trauma admissions involving orthopaedic procedures for 4 weeks in each of the three time periods. Data collected included age of the patient, date and time of admission, date of operation, mechanism of injury, operation performed, American Society of Anesthesiology (ASA) grade of the patient, grade of the operating surgeon, time in and time out of theater, length of surgery, and overall length of stay. Data for 2019 consisted of 108 cases, of which 14 records could not be used for length of surgery analyses due to missing data. In March 2020, 65 patient records were collected during this time frame, of which 8 records could not be used for length of surgery due to missing data. December 2020 yielded 77 records, of which 6 had some missing intraoperative time data sets. In one patient in 2019, the length of stay was subsequently removed because of a large time lag (> 1 month) between admission and operation due to transfers between hospitals. Intraoperative time was separately analyzed as length of surgery according to the type of surgery being performed, and the time under anesthesia.

When analyzing the length of surgeries, the surgeries performed were divided according to type into the following categories: open reduction and internal fixation (ORIF), hip hemiarthroplasty, intramedullary (IM) nail, dynamic hip screw (DHS), total hip replacement (THR), and other (which included combination surgeries and surgeries which were only performed in one of the two years and so could not be compared adequately).

### Data Analysis

Data was tested for normality using the Kolmogorov–Smirnov statistical test and was then analyzed through the use of unpaired, two-tailed *t* -tests when comparing means and the chi-squared test when comparing mechanisms of injury.

## Results

### Patient Demographics

**Fig. 1:**
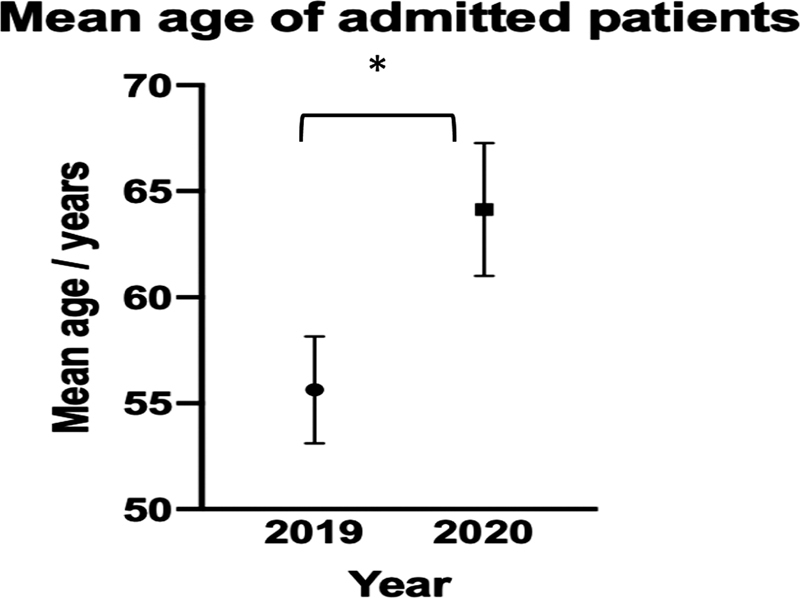
Mean age of all admitted patients. Error bars show ± standard error of mean (SEM). * indicates *p* < 0.05.

The mean age was compared for all study periods using a *t* -test, comparing two data sets at a time. The mean age of admitted patients was significantly different at *p* = 0.05, at 55.6 years in 2019 and 64.1 years in March 2020 ( *p* -value: 0.038022), and 67.7 years in December 2020 ( Fig. 1 ).

### Mechanism of Injury

**Fig. 2:**
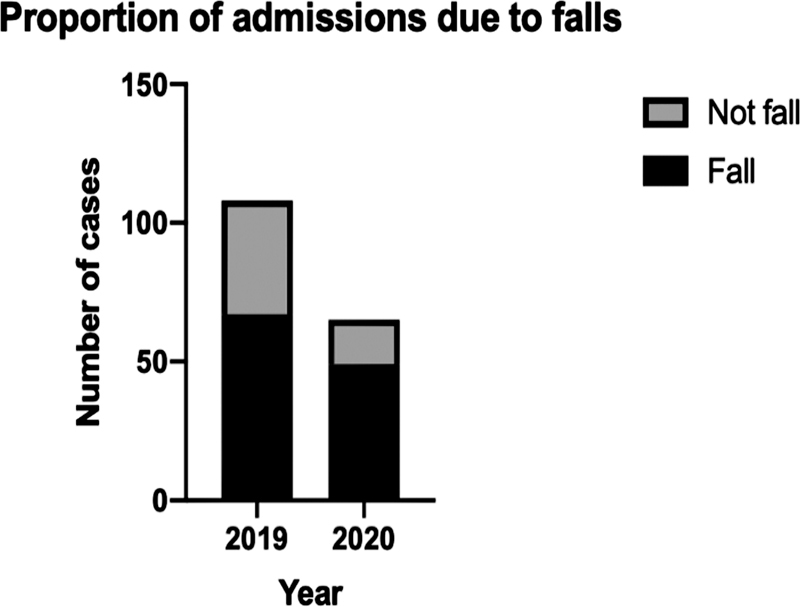
Mechanism of injury stratified by fall/not fall in 2019 and 2020.

In all study periods, the predominant cause of injury was falls. In 2019, 62% of the causes involved falling, and in March 2020, falls contributed to 75% of the total trauma operations undertaken, a difference of 13%. December 2020 was comparable to March, with 74% of injuries requiring an operation being secondary to falls. The chi-square results comparing the 2 years, however, showed this to be not significant ( *p* = 0.0793). The other mechanisms of injury were too diverse and uncommon to compare accurately between the two groups ( Fig. 2 ).

### Length of Stay

**Fig. 3:**
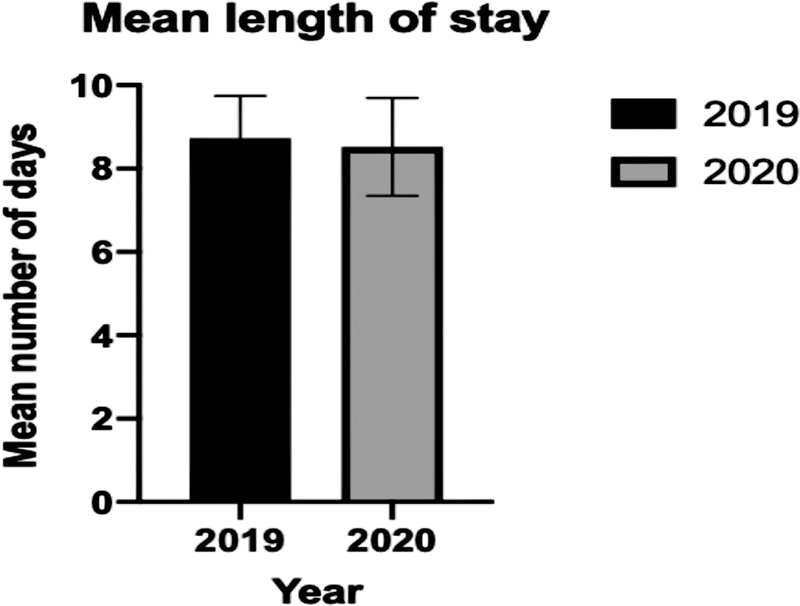
Mean length of stay for each year. Error bars show ± standard error of mean.

Mean length of stay did not vary significantly between the two initial periods, at 8.73 days in 2019 and 8.52 days in March 2020 ( *p* = 0.3843) ( Fig. 3 ).

**Fig. 4:**
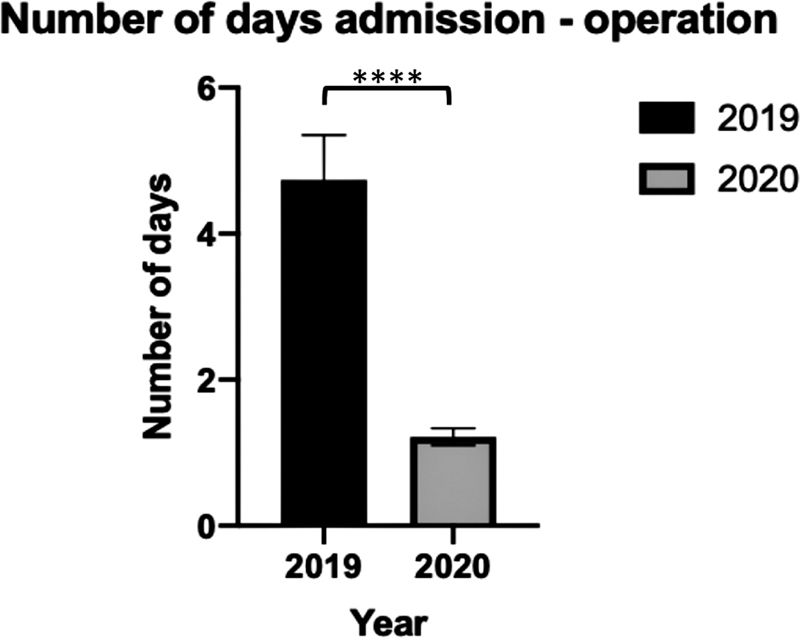
Mean length of delay between admission and operation. **** denotes *p* < 0.0001.

Time between admission and operation was, however, significantly different ( *p* = 1.23 × 10 ^−7^ ) ( Fig. 4 ).

### Type of Surgery

Types of surgery were categorized accordingly: ORIF, hip hemiarthroplasty, IM nail, DHS, THR, and other.

### Length of Surgery

**Fig. 5:**
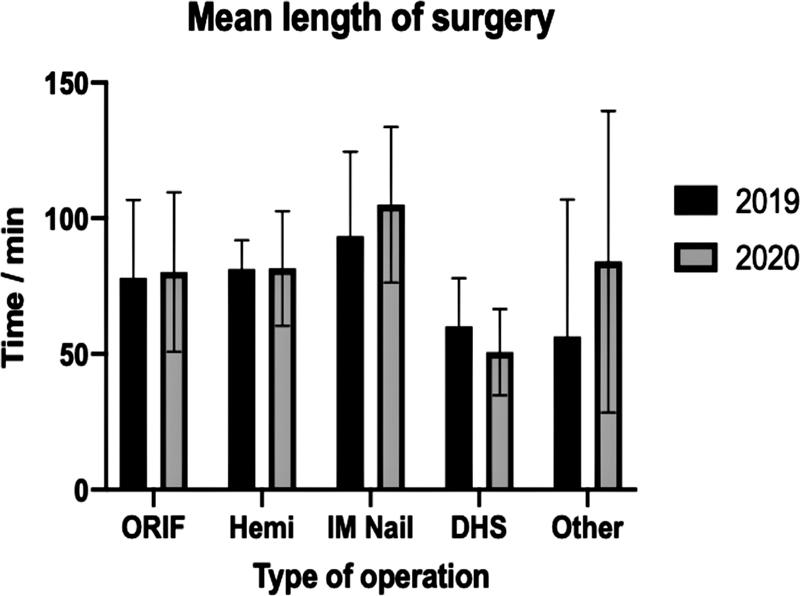
Mean length of operation for different surgeries in 2019 and 2020. Error bars show ± standard deviation. None of the results are statistically significant.

The surgeries performed were divided according to type into the following categories: ORIF, hip hemiarthroplasty, IM nail, DHS, THR, and other (which included combination surgeries and surgeries which were only performed in one of the two years and so could not be compared adequately) ( Fig. 5 ).

Neither stratified nor nonstratified mean length of surgery were statistically significant ( Table 1 ).

**Table 1:** Summary table of mean surgery length ± standard error of mean

Type of surgery	2019 (mean no. of min ± SEM)	2020 (mean no. of min ± SEM)	% change	*p* -Value
ORIF	78.07 ± 5.26	80.21 ± 7.84	+ 2.75	0.82 (ns)
Hemi	81.29 ± 4.03	81.54 ± 5.86	+ 0.31	0.97 (ns)
IM nail	93.40 ± 13.96	105 ± 11.73	+ 12.42	0.54 (ns)
DHS	60.20 ± 7.92	50.67 ± 5.30	–15.84	0.35 (ns)
THR	122.5 ± 7.5	−	−	−
THR MUA	12.5 ± 4.33	7 ± 2	–44.00	0.31 (ns)
Other	56.46 ± 8.30	84.00 ± 22.70	+ 48.78	0.30 (ns)

Abbreviations: DHS, dynamic hip screw; Hemi, hemiarthroplasty; IM nail, intramedullary nail; MUA, manipulation under anesthesia; ns, nonsignificant; ORIF, open reduction and internal fixation; SEM, standard error of mean; THR, total hip replacement.

Note: *p* -Values are given in the rightmost column. (There were no THR operations performed in 2020, hence the blank columns.)

### Time Under Anesthesia

**Fig. 6:**
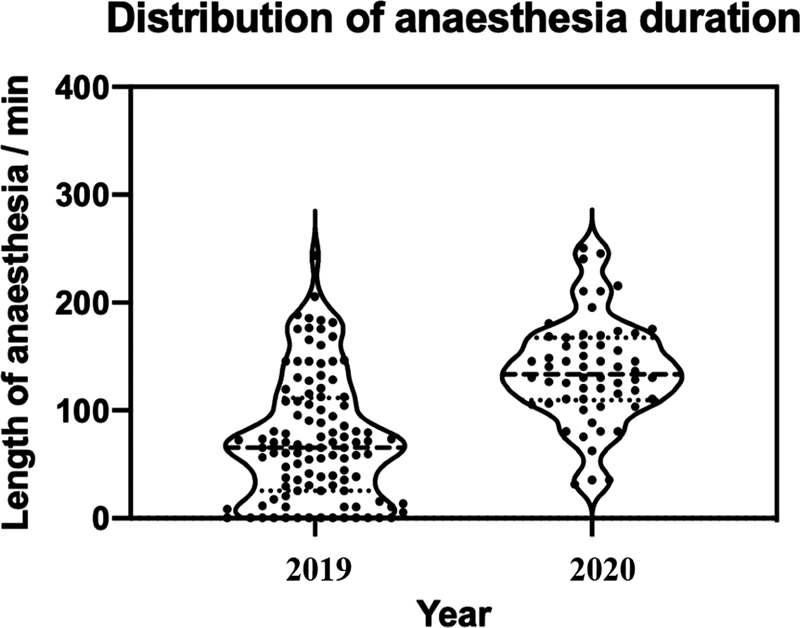
Violin plot showing the distribution of anesthesia length in 2019 and 2020. Data was adjusted to remove kernel density estimation below 0 minutes. The solid line represents the median, and the dashed represent the first and third quartiles.

The mean length of time under anesthesia in 2019 was 83 minutes, compared with 136 minutes in March 2020 ( *p* < 0.00001), a 63.8% increase. This further increased in December 2020 to 141 minutes. This had similar statistical significance in comparison to March 2019, but no significant change with March 2020 ( Fig. 6 ).

### ASA Grade of Patient

The mean ASA of patients in 2019 was 2.26. This increased to 2.35 and 2.36 in March 2020 and December 2020, respectively. The mode in all time points was ASA 3.

### Grade of Operating Surgeon

Grade of the operating surgeon was obtained from the electronic operation notes and the in-theater staffing documents. An operation was considered consultant delivered if the consultant surgeon was recorded as the primary surgeon on the operation note and as scrubbed in the theater staffing documentation. Operations where the consultant was not recorded as scrubbed were considered registrar (or in a handful of cases senior house officer) delivered. In 2019 43.5% of operations were consultant delivered, which dropped dramatically at the start of the pandemic to 26%, and again further in December 2020 to 17%.

Registrar delivered operations increased accordingly from 54.6 to 72 and 85% subsequently.

### COVID Status

COVID status for all patients in 2019 was negative. In the March 2020 cohort only one patient was identified as COVID positive prior to surgery. Nineteen were negative, and 44 were not tested for COVID-19 prior to undergoing surgery. Two of the untested patients were subsequently found to be positive on testing postsurgery.

In the December 2020 group all patients were swabbed on admission. Six were identified as COVID positive prior to undergoing surgery.

## Discussion

The COVID-19 pandemic presented several challenges to orthopaedic trauma service delivery. The redeployment of staff to provide ITU and HDU cover during this time led to a reduced workforce already strained by frequent sick leave due to quarantine measures. The added time required to don and doff personal protective equipment (PPE) as well as the changes in theater cleaning requirements all accumulated to lengthen the duration of procedures and limit the number of cases that could be done on a daily basis.

From the 23rd of March onwards, 4 6 lockdown was brought into effect and these strict lifestyle restrictions are reflected in the changes in the patient cohorts seen in 2020 as compared with the previous year. Our study has found that the mean age of admitted patients was significantly older during the pandemic than for the previous year. This is likely due to the restrictions which stopped all sports both at a competitive and recreational level as well as restrictions on parks which usually provides a proportion of our pediatric trauma. To reflect this, under 18s formed 10.28% of admissions in 2019 (11 patients), and only 3.13% of the admissions in 2020 (2 patients). In line with this the number of ASA 1 and 2 patients both dropped during the pandemic.

The lockdown rules in the U.K. restricted all nonessential travel, 4 which reduced the number of people commuting to work, particularly pertinent at a district hospital which serves suburbs as fewer patients travelled into London for work during this period. This could have resulted in fewer travel-related accidents, and as the workforce is predominantly a younger population, with around two-thirds under the age of 49, according to the Office of National Statistics, 7 in turn means that the mean age of patients seen at our DGH for the COVID time periods was higher. 8 This is compounded by the reduction in road traffic accident (RTA) cases directly as a result of the lockdown rules, with some studies showing a third of the number of RTA admissions during lockdown compared with in 2019. 9

As expected, the majority of injuries were caused by falls in all time points. The increase in proportion in 2020 from the previous year ties in with the previous observation of a higher mean age, as this demographic is at higher risk of fragility fracture 10 in a fall from standing.

Despite the greater emphasis placed on reducing hospital stays of trauma patients to minimize exposure to COVID-19, the mean length of stay remained unchanged throughout.

Preoperative delays were significantly reduced in March 2020. This is likely due to the reduced rate of admission requiring surgery with relatively well-preserved theater capacity. Pre-COVID trauma theater capacity consisted of a two-session dedicated trauma list daily, with the potential to utilize the elective and day-case theaters for trauma if space became available (i.e., on the day of cancellation). During the March lockdown there was no elective theater. One dedicated trauma theater ran on most days. However, there were 9 days during this period with no dedicated trauma list. Orthopaedic cases on these days were performed on a shared emergency list. During the December lockdown the dedicated trauma list ran every day except Christmas day. There were no days where staffing levels prevented this from occurring. Therefore, apart from 9 days during the March 2020 lockdown, trauma theater capacity was sustained at its prepandemic levels.

The fact that length of stay remained unchanged despite shorter times from admission to operation is most likely due to the population shift toward older patients with fragility fractures during this time. These elderly patients still require significant postoperative rehabilitation and there are limited areas where time can be reduced. This in turn is supported by the ASA data, which shows mean ASA increased from 2.16 pre-COVID to 2.35 and 2.36 for March and December 2020, respectively, with a much higher proportion of ASA 3 and 4 patients presenting during the pandemic. Second to this, rehabilitation facilities were likely reduced due to staff redeployment, affecting inpatient physiotherapy availability.

Increased postoperative stays could also be attributed to a greater incidence of pulmonary complications, as seen in patients with severe acute respiratory syndrome (SARS) coronavirus 2, who have a significantly worse outcome and mortality rate. 11 12 We recorded only patients who had a positive diagnosis of COVID-19 at the time of operation. Patients who subsequently developed COVID-19 while still an inpatient were not separately identified. One may expect that this could have occurred and have caused delay to discharge.

Length of surgery remained largely unchanged both when stratified for surgery type and as a whole. The Trust guidelines at the time allowed COVID-19 testing only for symptomatic patients on admission (cough or pyrexia). However, all orthopaedic patients were treated as suspected COVID positive and therefore full PPE was worn by all staff members for all cases, which added significant delays to time between cases. However, the clock start for operative time was taken as knife-to-skin, which occurred after PPE had been applied, resulting in the preservation of total operative time for each operation type when comparing between the time points.

Interestingly, to this point the grade of surgeon was significantly different prepandemic and during the pandemic. In the March 2019 cohort 43% of operations were consultant delivered, whereas March 2020 and December 2020 were 26 and 17%, respectively. We speculate that while consultants remained involved in the treatment decision-making process, the delivery was more registrar led, possibly as the consultants took on wider managerial responsibility in the department. Practically this meant supervising a full consultant led ward round of all patients every day.

The time that patients spent under anesthesia significantly increased during the pandemic. This is likely due to new anesthetic guidelines for COVID-19-positive patients requiring surgery, 13 including additional filter configuration, greater time for anesthetic staff to disinfect equipment before and after use, and donning and doffing of PPE by all staff entering and exiting the theater. This would have been particularly important in reducing exposure for the staff, as studies of the SARS outbreak have found staff performing intubations are at higher risk of being infected with the virus without sufficient use of PPE. 14 All patients requiring a general anesthetic were intubated rather than the use of a laryngeal mask airway (LMA), which again would increase the anesthetic time.

Patient logistical pathways and procedures in theater also changed as a result of the COVID-19 pandemic. 15 To avoid unnecessary contamination of surfaces and spaces patients were anesthetized and recovered directly in the operating theater, rather than the dedicated anesthetic room. Cleaning protocols were also changed as the theater was “rested” to allow virus particles to settle and a safe number of air exchanges to take place before cleaning of surfaces could begin. 16

Interestingly, this did not improve with time, as the December 2020 cohort had comparable anesthetic times to the March 2020 cohort. Certainly, this is multifactoral. 17 18 By December 2020 all patients were tested for COVID-19 on admission by polymerase chain reaction test from a nasopharyngeal swab. Those who had a negative test within 72 hours of surgery did not require full PPE to be applied by staff and use of an endotracheal tube or LMA was left to the clinical decision of the anesthetist. One would have expected these changes to improve anesthetic time, whereas instead it slightly increased. Unfortunately, it was not possible to ascertain the grade of the anesthetist from our electronic record system, or whether the patient had a regional block in addition to general anesthetic. Certainly, in March 2020 the focus was to minimize exposure to patient and staff at all points, and therefore use of regional anesthesia as an adjunct may have reduced, However, we could not substantiate this.

As a single-center study, the sample size did not allow for detailed statistical analyses; expanding the study over multiple district hospitals would more accurately determine trends, if any. Furthermore, as a retrospective study, some statistics could not be extracted, which would have provided a more detailed analysis of the contributing factors.

## Conclusion

Orthopaedic trauma remains an essential service which has always had to overcome the challenges of capacity and resources in busy cities like London. Despite the reduction in trauma volume during the COVID-19 lockdown, there have still been significant pressures on the health care system due to new challenges in the face of this new disease.

Despite the unprecedented burden on our Trust, we have still been able to provide timely and effective care for our trauma patients. The result of new guidelines and the logistical pressures the pandemic placed on the service led to a reduction in admission-operation times but greater time being spent on anesthesia. By understanding the effects of the lifestyle restrictions brought about by a national or local lockdown on trauma services as well as the impact of COVID-19 on service delivery measures such as length of surgery and stay, health care managers can plan for service delivery in the future as we attempt to return to nonemergency orthopaedic services and move lockdown restrictions are eased.
